# Resource acquisition and allocation traits in symbiotic rhizobia with implications for life-history outside of legume hosts

**DOI:** 10.1098/rsos.181124

**Published:** 2018-12-12

**Authors:** Katherine E. Muller, R. Ford Denison

**Affiliations:** 1 Graduate Program in Plant and Microbial Biology, University of Minnesota, St Paul, MN 55108, USA; 2 Department of Ecology Evolution and Behavior, University of Minnesota, St Paul, MN, USA

**Keywords:** symbiosis, nitrogen fixation, dormancy, life-history, plant–microbe interactions, polyhydroxyalkanoate

## Abstract

Resources that microbial symbionts obtain from hosts may enhance fitness during free-living stages when resources are comparatively scarce. For rhizobia in legume root nodules, diverting resources from nitrogen fixation to polyhydroxybutyrate (PHB) has been discussed as a source of host–symbiont conflict. Yet, little is known about natural variation in PHB storage and its implications for rhizobial evolution. We therefore measured phenotypic variation in natural rhizobia populations and investigated how PHB might contribute to fitness in the free-living stage. We found that natural populations of rhizobia from *Glycine max* and *Chamaecrista fasciculata* had substantial, heritable variation in PHB acquisition during symbiosis. A model simulating temperature-dependent metabolic activity showed that the observed range of stored PHB per cell could support survival for a few days, for active cells, or over a century for sufficiently dormant cells. Experiments with field-isolated *Bradyrhizobium* in starvation culture suggest PHB is partitioned asymmetrically in dividing cells, consistent with individual-level bet-hedging previously demonstrated in *E. meliloti*. High-PHB isolates used more PHB over the first month, yet still retained more PHB for potential long-term survival in a dormant state. These results suggest that stored resources like PHB may support both short-term and long-term functions that contribute to fitness in the free-living stage.

## 1. Introduction

Host organisms often provide microbial symbionts with a rich supply of resources in an otherwise resource-scarce environment. Many bacteria accumulate carbon and energy in polyhydroxybutyrate (PHB) and similar compounds during interactions with eukaryotic hosts [1,2], including nitrogen-fixing rhizobia in legume root nodules [3], bacteria in plant rhizospheres [4], bioluminescent bacteria in squid light organs [5], endophytic bacteria in marine sponges [6] and *Legionella* bacteria in freshwater amoebae [7]. Once symbiotic microorganisms exit hosts, stored PHB can enhance their fitness during free-living life-history stages by supporting survival or reproduction during starvation [4,8,9], protecting against abiotic stressors [10–12] and facilitating transitions in and out of dormancy [13]. If PHB does play an important role in the fitness of free-living microorganisms, then the persistence of heritable variation within populations in the amount of PHB stored during symbiosis would raise questions about evolutionary trade-offs or life-history strategies that vary in their dependence on stored resources during the free-living stage.

For symbiotic rhizobia, it has been hypothesized that the evolution of traits influencing the amount of PHB stored during symbiosis would be shaped by opposing selection pressures in the free-living and symbiotic stages [8,14]. Rhizobia that accumulate more PHB in symbiosis would (in theory) have higher fitness after they exit root nodules, leading to selection during the free-living stage favouring strains that accumulated more PHB during symbiosis. However, because PHB accumulation draws from a limited supply of metabolic resources that could otherwise be used for nitrogen fixation [15], rhizobia that prioritize allocation to PHB over nitrogen fixation may induce the host plant to redirect resources to more-beneficial nodules (i.e. host sanctions [16]), possibly leading to selection against greater PHB accumulation during the symbiotic stage. There is sound experimental evidence supporting the physiological role of PHB as a source of energy for free-living rhizobia [8] and as a sink for resources that would otherwise be used for nitrogen fixation [17,18], although further research is needed to understand how the physiological cost of PHB affects host-plant fitness.

Several key empirical gaps must be filled to connect the physiological functions of PHB with evolutionary hypotheses about selection in the free-living and symbiotic stages. One key gap is the extent to which natural rhizobia populations contain heritable phenotypic variation in the amount of PHB acquired during symbiosis (non-heritable and non-varying traits do not contribute to evolution by natural selection). Experiments with mutant rhizobia strains show several genetic pathways could create heritable variation in the amount of PHB that rhizobia acquire during symbiosis [17–21]. However, the heritable phenotypic variation has not been evaluated in natural populations. Another key gap is our understanding of how heritable variation in PHB storage contributes to fitness differences among rhizobia. The hypothesis that being able to store more PHB during periods of high carbon availability in root nodules or in the rhizosphere would enhance fitness in the free-living stage makes intuitive sense and is supported by experiments with high- and low-PHB populations of the same rhizobia strain generated through culture conditions or centrifugation [8]. However, fitness differences due to non-heritable (experimentally manipulated) phenotypic variation within a genotype may not predict fitness differences due to heritable phenotypic variation among genotypes. One reason is that rhizobia genotypes that differ in PHB accumulation may also differ in other life-history traits related to how they allocate stored resources over time and among life functions. For example, many non-spore-forming bacteria (like rhizobia) can lower their energy requirements by entering a reversible resting metabolic state of dormancy [22]. Dormancy is widespread among soil bacteria [23] and enables some microorganisms to survive for thousands of years [24]. There is evidence that co-occurring soil microbes can vary widely in their level of metabolic activity during dormancy, consistent with different life-history strategies prioritizing competitiveness or survival [25,26]. Organisms that maintain higher resting metabolic activity respond to new resources more quickly [27], probably improving their ability to compete for sporadic resources while also sacrificing some of the survival benefits of deep dormancy. While previous studies on soil microbial communities describe dormancy variation at the phylum level [25,26], research on natural *Escherichia coli* isolates shows that dormancy traits can vary substantially among conspecific bacteria [28]. Therefore, it is feasible that rhizobia populations could contain heritable phenotypic variation in dormancy traits (such as resting metabolism) as well as PHB accumulation, which would complicate predictions about genetic covariance between the amount of PHB acquired during symbiosis and fitness during the free-living stage. Thus, experimental manipulation of PHB in a single rhizobia genotype may over-simplify the selection pressures on PHB accumulation in wild populations by excluding variation in other life-history traits, including dormancy.

This study fills essential gaps needed to connect what we know about the physiological functions of PHB with evolutionary hypotheses about the role of PHB in the symbiotic and free-living stages of nitrogen-fixing rhizobia. The first part assesses naturally occurring phenotypic variation in the amount of PHB acquired during symbiosis, using rhizobia populations associated with an agricultural host (*Glycine max*) or a wild host (*Chamaecrista fasciculata*). We evaluated the extent of naturally occurring phenotypic variation (which includes variation due to both genotype and environment) by measuring PHB in nodules sampled from plants growing in the field or inoculated with field soil. We evaluated the genetic component of natural phenotypic variation by conducting experiments to measure apparent broad-sense heritability in a sample of field-isolated rhizobia (apparent because not all isolates were genotyped). The second part evaluates potential effects of heritable variation on the fitness of rhizobia during the free-living stage using a combination of (i) modelling to simulate PHB use under different resting metabolic rates and (ii) starvation experiments to observe PHB use and population persistence of rhizobia extracted from soya bean nodules. While our focus is on PHB accumulation in nodules, we recognize that rhizobia could also accumulate PHB in the free-living stage using carbon from root exudates or decaying roots [1]. It is feasible that traits underlying variation in PHB accumulation within root nodules would also mediate PHB accumulation in the rhizosphere, though that question is beyond the scope of this study.

## 2. Material and methods

### 2.1. Measuring PHB accumulation with flow cytometry

We quantified the PHB phenotype as PHB per cell for thousands of rhizobia extracted from a single nodule. Assuming mixed nodules are rare [29], the range of PHB per cell within a nodule usually represents phenotypes for a single rhizobia strain. Even within mixed nodules, the mean PHB per cell probably represents the phenotype of the dominant strain within the nodule [29].

We measured PHB per cell using flow cytometry, based on methods described by Ratcliff *et al*. [8]. We estimated PHB per cell from flow cytometry measurements using a set of six archived *Ensifer meliloti* standards (strain 1021) with varying PHB per cell (induced by culture conditions) previously measured with gas chromatography [8]. Within 48 h of harvest, nodules were surface-sterilized by rinsing in 95% ethanol, soaking for 5 min in 30% bleach and rinsing five times in sterile deionized water. Nodules were crushed in sterile phosphate buffer saline (PBS) and liquid extracts were fixed in 30% ethanol for 30 min, centrifuged at 5000*g* for 5 min, resuspended in PBS, then stored at 4°C. Shortly before flow cytometry, fixed nodule extracts were diluted 10-fold in PBS to 10^5^ to 10^7^ rhizobia ml^−1^ (depending on the size of the nodule) and stained in a 1 µg m^−1^ l solution of a quantitative lipid stain, either Nile Red or Bodipy 505/515 [30].

We assayed PHB in stained rhizobia samples with a Benton Dickson FACSCalibur flow cytometer, which measures fluorescence and forward scatter (a proxy of cell size) for thousands of rhizobia cells in each sample. We estimated PHB per cell based on fluorescence of the quantitative lipid stain measured on the FL1 channel (for Bodipy 505/515) or the FL3 channel (for Nile Red) of the flow cytometer. All six *E. meliloti* standards were stained and run on the flow cytometer concurrently with each batch of samples to control for day-to-day variation.

The software FlowJo (v. 7.6.1) was used to exclude debris and calculate the geometric means of FL1 or FL3 fluorescence and forward scatter of rhizobia cells in each sample. Geometric means were used to reduce the influence of outliers on estimates of PHB per cell. For each sample, we excluded debris from flow cytometry data by selecting clouds of cells with similar size and shape (based on plots of forward scatter and side scatter) and excluding cells with near zero FL1 or FL3 fluorescence (based on histograms, electronic supplementary material, appendix A). The geometric-mean fluorescence for cells sampled from each nodule was converted to mean PHB per cell (pg) using a standard curve from the *E. meliloti* standards.

### 2.2. Nodule samples from field and trap plants

Nodules were collected from plants growing in the field (*G. max* and *C. fasciculata*) or plants inoculated with field soil and grown in the greenhouse (*G. max,* cultivar MN-0095). Because rhizobia were not genotyped, these samples do not provide direct information about what proportion of phenotypic variation was due to heritable differences among rhizobia genotypes (and therefore subject to natural selection). Instead, they provide information about the extent to which PHB accumulation by rhizobia varies among nodules within a host-plant population at a single location and time point. Previous studies on rhizobia population structure in agricultural systems have found that a single host plant typically hosts between 4 and 14 different rhizobia genotypes across different nodules [31–33].

#### 2.2.1. Glycine max

Field-collected soya bean nodules came from *G. max* cultivars S09Ry64 and Dyna-Gro planted in May 2015 within a 20 × 10 m field plot in the Sand Plain Research Farm in Becker, MN (with permission from on-site staff). In mid-July, when plants reached the mid-pod-filling stage, we collected 88 nodules from 16 plants distributed across 4 subplots (approx. 5 nodules per plant, with 4 plants sampled from each plot). To minimize PHB catabolism, nodules were stored on ice during fieldwork, then refrigerated at 4°C for 24–72 h before processing. Fixed samples were stored at 4°C for approx. 2 months before being stained with Nile Red and assayed for PHB.

Although some subplots were inoculated, soya bean nodules in the Midwest US are rarely dominated by inoculum strains [34] and we suspect that this was true at Becker. Each subplot sampled in the Becker site represented a different inoculation treatment intended to compare effects of three rhizobia strains previously found to differ in PHB accumulation during pilot experiments (applied as liquid inoculum with approximately 10^7^ cells per seed). Each subplot was inoculated with either a high-PHB field isolate, a low-PHB field isolate, a widely used beneficial rhizobia strain with low-to-moderate PHB accumulation (*Bradyrhizobium diazoefficiens* USDA110), or diluted sterile culture media as the uninoculated control. We initially chose this site because it had no known history of soya bean and we expected the soil to contain low numbers of resident rhizobia. However, several factors suggest that the population size of resident rhizobia was high enough to outcompete our inoculum strains. First, plants in the uninoculated subplots showed no signs of nitrogen deficiency (i.e. yellowing leaves or reduced growth) or reduced nodulation (based on visual inspection of roots), which suggests that the supply of nitrogen from rhizobia was similar between inoculated and uninoculated subplots (no nitrogen fertilizer was applied). Second, PHB per cell varied widely among nodules from the same subplots and did not differ significantly between subplots with different inoculation treatments, according to an ANOVA (*F* = 1.01, *p* = 0.39, residual d.f. = 84; electronic supplementary material, appendix A). Previous studies have found that rhizobia applied as inoculum typically occupy a minority of nodules unless resident rhizobia populations are very low—i.e. low enough to produce signs of nitrogen deficiency in uninoculated areas [35–38]. Therefore, it is likely that resident rhizobia made up a large portion of our field-sampled soya bean nodules, although we cannot make firm conclusions about the identity of rhizobia in our sample without genotyping data.

We also assessed phenotypic variation in a larger sample of 1276 soya bean nodules obtained from 160 soya bean trap plants (cultivar MN-0095) grown in the greenhouse and inoculated with soil from 40 field plots. The soil was collected from agricultural plots at the Southern Research and Outreach Center in Waseca, MN (with permission from on-site staff) as part of an experiment comparing the distribution of nodule PHB under different long-term crop rotations, which will be presented in a future paper along with a detailed description of the field plots and sampling methods. For the purposes of this study, each plot can be considered an independent replicate capturing spatial variation in resident rhizobia. The management of these plots has included chemical fertilizers, herbicides and conventional tillage, with no rhizobia added through inoculation [39,40]. We bulked together 10 soil cores from each plot and inoculated four replicate plants per plot with soil slurries mixed separately for each plant. Each soil slurry contained 20 g of field soil diluted in 80 ml of saline solution (0.85% sodium chloride) and was prepared as described by Bala *et al*. [41]. Soya bean seeds were surface-sterilized and sown in pots made of stacked Magenta-box units filled with a 1:1 mixture of sand and vermiculite, then autoclaved [42]. Germinated seeds were inoculated with 1 ml of soil slurry. Plants were grown in the greenhouse and fertilized and watered with an N-free nutrient solution [43] through a cotton wick from the bottom of the growth unit [42]. Plants in the greenhouse were randomly distributed into blocks of 12–16 plants on a single tray, including at least one uninoculated control. None of the uninoculated plants produced any nodules, which indicates that cross-contamination among plants was minimal. Once plants reached the early pod-filling stage, we harvested eight nodules per plant, extracted rhizobia from surface-sterilized nodules and fixed nodule extracts in 30% ethanol within 48 h. Fixed samples were stained with Bodipy 505/515 for measuring PHB. We retained a portion of live (unfixed) rhizobia extract from each nodule for further isolation (archived at −80°C in 25% glycerol). We also weighed dried shoots and nodules to assess any confounding variation due to host plant size.

#### 2.2.2. Chamaecrista fasciculata

We collected a total of 27 nodules from 13 locally sourced *C. fasciculata* that we planted in a prairie restoration within the Grey Cloud Dunes Scientific and Natural Area in Cottage Grove, MN. The restoration has been maintained by the Minnesota Department of Natural Resources (MN-DNR) since 1998 and rhizobial inoculum has not been applied during that time. Permission for fieldwork was obtained through a permit from the MN-DNR, issued to Ruth Shaw. In June 2017, we planted 100 seeds (combined from 20 maternal lines sourced from Grey Cloud Dunes) at 1 m intervals across two parallel 50 m transects spaced 2 m apart. Seeds were scarified and surface-sterilized with 50% bleach before planting. Nodules and above-ground parts were harvested in early September when most plants had produced flowers, but not fruits. Owing to low germination rates, we were only able to obtain 27 nodules from 13 plants scattered throughout the two transects (we could only find one or two nodules on most plants). Fixed nodule extracts were stained with Bodipy 505/515 to measure PHB. We also weighed dried shoots, weighed fresh nodules and used dilution plating to estimate rhizobia per nodule. For most samples, we spread diluted nodule extract onto Arabinose-Gluconate (AG) agar [44], as well as AG agar supplemented with cycloheximide (200 µg ml^−1^) and Brilliant Green (BG) (1 µg ml^−1^) to exclude likely contaminants [45]. Dilution plates for 13 out of the 27 nodules showed growth on plain AG agar and no growth on supplemented AG agar, which suggests that these rhizobia were inhibited by BG. Based on previous studies showing variation in BG sensitivity among *Bradyrhizobium* taxa [45], we interpreted tolerance and sensitivity to BG inhibition as a taxonomic character trait distinguishing at least two distinct genetic groups of rhizobia within our sample of *C. fasciculata* nodules. We did not collect the genotyping data needed to conclude whether BG-tolerant or -sensitive rhizobia from different nodules represented different rhizobia strains.

### 2.3. Experiments to assess heritability in PHB variation

#### 2.3.1. Rhizobia isolates

**Figure 1.**
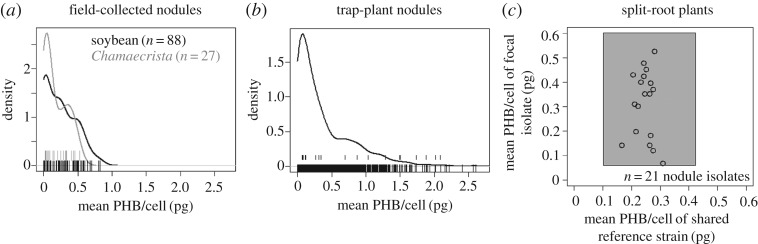
PHB accumulation varies widely among co-occurring rhizobia. Phenotypic distribution PHB per cell averaged within nodules collected from (*a*) *G. max* and *C. fasciculata* growing in the field, and (*b*) *G. max* inoculated with field-collected soil. The *y*-axis shows the probability density of PHB measurements estimated from a Gaussian kernel function in R [46]. (*c*) Variation in PHB accumulation due to environment only (horizontal axis) or genotype plus environment (vertical axis), measured in split-root *G. max* plants inoculated with a field-isolated strain (upper row of rug plot in (*b*)) paired with a common reference strain on the other half of the root system. Each point is mean of nine replicate nodules each of focal and reference strains, sampled from three plants. The grey box is the empirical 95% confidence interval for all nodules, which incorporates variability due to genotype, environment and measurement error.

We estimated the broad-sense heritability of PHB accumulation in two sets of rhizobia isolates from our field site in Waseca, MN. The first set of 20 isolates was obtained in 2014 from soya bean nodules haphazardly sampled from plants growing in the field. Nodules from the field were surface-sterilized and used to inoculate soya bean plants (cultivar MN-0095) in aseptic growth pouches. Isolates were cultured from single colonies obtained by streaking surface-sterilized nodules from trap plants on AG agar. Single-colony cultures were archived at −80°C in 25% glycerol. DNA fingerprinting with the BOX-AR1 primer confirmed that all 21 isolates were genetically distinct rhizobia strains (those data will be presented in a future paper). The second set of 20 rhizobia isolates comes from our collection of 1276 trap-plant soya bean nodules used to assess naturally occurring phenotypic variation in PHB accumulation. Rhizobia from 20 nodules were selected to cover the range of phenotypic PHB variation shown in figure 1*b*. Archived extracts from surface-sterilized nodules were streaked onto AG agar and single colonies were cultured in liquid AG medium and archived at −80°C in 25% glycerol. Genotyping data on these isolates (including 16S rRNA sequencing) will be presented in a future paper with molecular work on PHB-related genes. Preliminary data show that these 20 isolates represent at least three taxonomic groups of *Bradyrhizobium* distinguishable based on 16S sequence variation (the 16S regions we have sequenced so far are not suitable for distinguishing genotypic variation below the species level). We recognize that our isolation methods do not meet the recommended purity standards typically used for isolating rhizobia from nodules (i.e. re-streaking single colonies several times before reisolating from trap-plant nodules). Even so, we can say that our sample of rhizobia isolates represents a set of phenotypically variable, and probably genetically variable, *Bradyrhizobium* from a single field site.

#### 2.3.2. Growing conditions

We conducted two separate experiments to measure apparent broad-sense heritability in PHB accumulation in each set of rhizobia isolates. Both experiments used soya bean plants (cultivar MN-0095) sown in aseptic growth pouches (using surface-sterilized seed), fertilized with an N-free nutrient solution [43] and placed in a growth chamber set at 26°C with a 12 h light cycle. Both experiments included a set of uninoculated controls to detect cross-contamination (neither set of uninoculated controls produced nodules).

The first experiment used the set of 20 isolates from field-collected nodules, plus the inoculum strain *B. diazoefficiens* USDA110. Each rhizobia isolate/strain was used to inoculate three replicate soya bean plants sown in 16 × 18 cm grouch pouches (each plant receiving approximately 5 × 10^6^ rhizobia from a single isolate/strain). We harvested nodules at the early stages of leaf senescence and measured PHB in three nodules per plant using the lipid stain Nile Red and a DNA stain (SYTO 13) to help exclude debris [47]. (We removed the DNA stain from later protocols because it was not especially helpful and added extra labour.) The estimates of the broad-sense heritability among isolates were based on PHB measurements from nine nodules per isolate, collected from three replicate plants.

The second experiment used the set of 20 isolates from trap-plant nodules (figure 1*b*). Instead of using one rhizobia isolate per plant as in the first experiment, we measured PHB variation among isolates in split-root plants (three replicate plants per isolate) inoculated with the focal isolate on one side of the root system and a common reference strain on the other side of the root system [48]. Our rationale for using split-root plants was to (i) provide a measurement of PHB variation more relevant to the field, where plants generally host multiple strains of rhizobia and (ii) to reduce non-heritable variation in PHB due to feedbacks between nitrogen fixation and total carbon supply for rhizobia [49]. The reference strain was one of the 2014 field isolates from the first experiment, which we selected because it showed growth and nodulation capabilities similar to USDA110 but without hydrogen uptake, which interferes with nitrogen-fixation measurements [50] that were part of a separate study. Surface-sterilized soya bean seeds were sown in large, divided growth pouches (25 × 21.5 cm) and radicles were trimmed to induce root branching when they reached a length of approximately 1 cm. Each side of the growth pouch was inoculated with approximately 5 × 10^6^ rhizobia diluted in 10 ml of sterile water. Once plants reached the mid-to-late pod-filling stage, we collected three nodules from each side of the divided pouch to measure PHB with Bodipy 505/515. Owing to plant mortality, seven of 20 isolates had fewer than three plant replicates, leaving three to six replicate nodules per focal isolate per side instead of nine.

### 2.4. Statistics

We used R v. 3.4.2 [46] for all statistical analyses and modelling. To estimate the apparent broad-sense heritability of PHB accumulation in split-root plants and singly inoculated plants, we obtained variance components from a linear model with nodule mean PHB per cell explained by random effects of isolate and plant replicate, using the package lme4 in R [51]. We estimated the broad-sense heritability by dividing the variance among isolates by the total phenotypic variance (i.e. the sum of variance among isolates, variance among plant replicates and residual variance within plants) [52]. We used bootstrap resampling (randomly resampling plants 10 000 times with replacement) to obtain mean estimates and 95% confidence intervals for the broad-sense heritability [53]. We tested the statistical significance of heritability estimates against the null hypothesis that the apparent broad-sense heritability could be due to environmental differences among plants unrelated to the identity of rhizobia. We rejected the null hypothesis if the observed broad-sense heritability was greater than 95% of null heritability estimates generated by randomly reassigning plants to isolates (10 000 permutations).

We also used a permutation test (10 000 permutations) to assess whether the taxonomically variable trait we identified in rhizobia from *C. fasciculata* nodules (sensitivity to Brilliant Green, or BG) showed any relationship with PHB phenotype. Because nine of the nodules with BG-sensitive rhizobia came from the same plant, and most plants only had one or two nodules, we used the average PHB measurement for BG-sensitive and BG-tolerant nodules within each plant to reduce pseudoreplication.

### 2.5. Metabolic model of PHB use as a function of temperature

Our model estimates the amount of PHB per day required to support an individual rhizobia cell in a resting state with varying levels of metabolic activity. Two limitations of this approach are that it does not incorporate effects of PHB on reproduction, which have been described previously [8], or incorporate resource inputs from the rhizosphere. Our intention was not to exactly simulate rhizobia in natural conditions but to explore how variation in resting metabolic rate over a biologically feasible range could mediate the effects of PHB per cell on the fitness of rhizobia during the free-living stage.

To estimate how long a rhizobia cell could benefit from PHB acquired during symbiosis, we simulated the metabolic requirements of a rhizobia cell in the soil by combining parameters of temperature-dependent metabolism from Price & Sowers [22] with a function simulating seasonal changes in soil temperature [54], which we fit to soil temperature data from the Southern Research and Outreach Center in Waseca, MN (electronic supplementary material, appendix B). Price & Sowers [22] defined two sets of metabolic parameters for non-growing bacteria based on distinct metabolic states proposed by Morita [55] and published measurements of prokaryote metabolism for a variety of organisms and communities from ecological samples, including agricultural soil. We used ImageJ with Price & Sowers' figure 1 (a plot of log-scale metabolic rate versus temperature extrapolated from many published measurements) to obtain slopes and intercepts for calculating temperature-dependent metabolic rates for bacteria in a state of full somatic maintenance (i.e. all active cell functions except new biomass production) or deep dormancy metabolism (i.e. minimal energy expenditure for molecular repair) in response to temperature. Their metabolic rates for full somatic maintenance and dormancy span a 10^3^-fold range, which we treated as a floor and a ceiling for resting metabolism in rhizobia (acknowledging that these published rates come from a wide range of prokaryotes in various ecosystems). We also considered three intermediate metabolic levels between full somatic maintenance and deep dormancy (10%, 1% and 0.1% of full somatic maintenance, table 1).

**Table 1.** Metabolic coefficients. The coefficients for full somatic maintenance (FSM) and dormancy come from figure 1 of Price & Sowers [22]. The terms *α* and *β*, respectively, represent the log-transformed metabolic rate as a function of temperature in Celsius: log_10_ (*m*) = *α* T + *β*, where *m* is the metabolic rate (g C respired per g C in structural biomass per day) and *T* is the soil temperature (in Celsius).

metabolic state	*α*	*β*
full somatic maintenance (FSM)	9.9 × 10^2^	−5.14
intermediate (10% of FSM)	9.9 × 10^2^	−6.14
intermediate (1% of FSM)	9.9 × 10^2^	−7.14
intermediate (0.1% of FSM)	9.9 × 10^2^	−8.14
dormancy	8.4 × 10^2^	−8.17

The model calculates how much PHB a rhizobia cell would require to support its metabolic needs for *t* days, based on a per-day metabolic rate described in table 1.

2.1 $$\text{PH}{\text{B}}_{t}=\left[\overset{t}{\underset{1}{\sum }}(m\times bC_{b})\times {\left(C_{\;phb}\right)}^{-1}\right].$$

The amount of PHB used each day (calculated as pg) is proportional to the metabolic rate (*m*), which varies in response to temperature and can be set to represent different levels of metabolic activity using the coefficients in table 1. The metabolic rate is the amount of carbon that a cell must respire to support resting metabolism, per gram of biomass carbon, for 1 day (g C g^−1^ biomass C day^−1^). A cell's biomass carbon is its structural biomass (*b*), not including PHB, multiplied by a stoichiometric conversion factor for biomass carbon (*C_b_*, g C per g of structural biomass) that assumes bacterial biomass has an average chemical composition of C_8_H_8_O_2_N_1_ [56]. The cell's structural biomass (*b*) is held at a constant 0.5 pg, based on a range of approximately 0.2–0.5 pg previously described for *Bradyrhizobium* bacteroids [57]. The amount of carbon used per day is converted to the amount of PHB used per day with another stoichiometric conversion factor (*C_phb_*, g C per g PHB), which assumes that PHB has an average chemical composition of C_4_H_6_O_2_ [56,58].

We simulated seasonally fluctuating soil temperature (*T*) with a first-order Fourier equation [54] fit to monthly soil temperature data from the Southern Research and Outreach Center (Waseca, MN) where we obtained most of our rhizobia. We fit the equation to monthly average soil temperatures from 2010 to 2014, measured at a 20 cm depth. Because soil temperatures below approximately 15 cm remain relatively stable compared to surface temperatures, they can be adequately approximated without diurnal oscillation [54]. We used the equation to extrapolate daily soil temperatures from monthly averages.

2.2 $$T_{d}=\frac{1}{2}(T_{\max}+T_{\min})+\frac{1}{2}(T_{\max}-T_{\min})\text{sin}\left(\frac{2\pi }{365}(d-\delta )\right).$$

In equation (2.2), *d* is the calendar day (between 1 and 365), *T*_max_ and *T*_min_ are the maximum and minimum annual temperature, and *δ* is the calendar day on which the temperature reaches the midpoint between *T*_max_ and *T*_min_. *T*_max_ and *T*_min_ were set to 25°C and −3°C, which were the average maximum and minimum temperature for the years 2010 through 2014, excluding an unusually cold winter in 2012. We set the midpoint date to 5 May (*δ* = 121) based on the visual fit of the equation line to the temperature data (electronic supplementary material, appendix B). While this approach does not capture temperature variability within days or among years, it approximates the seasonal temperature conditions that would be experienced by a free-living rhizobia cell in southern Minnesota soils.

### 2.6. Starvation cultures

To better understand how rhizobia use PHB after leaving nodules, we compared starving populations of nine rhizobia isolates plus one inoculum strain (*B. diazoefficiens* USDA110), which varied in initial PHB per cell due to heritable differences in PHB accumulation during symbiosis (measured in the single-inoculation experiment described above). The set-up and materials for our starvation experiment were identical to those described by Ratcliff *et al*. [8], except that we used rhizobia extracted from nodules instead of cultured rhizobia.

Rhizobia populations for the starvation culture experiment were extracted from nodules collected from singly inoculated soya bean plants (cultivar MN-0095) sown in growth pouches and grown in conditions identical to those described for the heritability experiments. For each isolate, we initiated starvation cultures with rhizobia populations extracted from pooled nodules from three mature plants. Starvation cultures contained rhizobia extracted from nodules surface-sterilized with bleach and diluted to approximately 10^5^ cells ml^−1^ (determined with OD600) in a starvation buffer made of carbon-free M9 medium supplemented with 0.1 g l^−1^ thiamine to enable carbon metabolism and made with double-deionized water (Ratcliff *et al*. [8]). Before starvation, nodule extracts were double washed with starvation buffer to remove residual carbon. We also removed residual carbon from glassware by washing it in 0.6 M HCl and pyrolysing it at 500°C for 4 h. To prevent evaporation and avoid introducing organic volatiles during long-term sampling, we distributed starvation cultures into 1 ml aliquots enclosed in sterile 2 ml microcentrifuge tubes. Tubes were shaken daily during the first month and weekly thereafter. For two isolates, we made an additional set of starvation cultures at a higher population density (approx. 10^6^ cells ml^−1^). In the soil, nodulating rhizobia can have a wide range of densities, usually between 10 and 10^7^ cells g^−1^ soil [34,59].

We sampled tubes of starvation culture weekly for the first month, then again after 4 and 15 months to monitor population size and PHB use. We diluted a portion of starvation culture from each tube to measure rhizobia population size (using dilution plating on AG agar), then fixed the remaining portion in 30% ethanol and stored it at −80°C in 25% glycerol so that samples collected at different times could be assayed together for PHB, as described above. Dilution plating showed that some starvation cultures contained contaminants, probably present in nodules or introduced during processing. Because removing replicates with substantial contamination removes most of our data and does not qualitatively change results (electronic supplementary material, appendix E), we present results for all samples with countable rhizobia, acknowledging that contaminants probably add noise. Additional details on methods and contaminants are included in the electronic supplementary material, appendix E.

## 3. Results

### 3.1. Phenotypic distribution of PHB accumulation in natural rhizobia populations

Our measurements show extensive phenotypic variability in PHB within three samples of rhizobia measured in nodules collected from the field (soya bean and *C. fasciculata,* figure 1*a*) or from trap plants inoculated with field soil (soya bean, figure 1*b*). The shape of the PHB distribution was qualitatively similar across all three samples from two host plants, showing a right-skewed probability density with a small minority of nodules showing little-to-no PHB accumulation and a larger minority showing much higher PHB accumulation than average (figure 1*a,b*). The range of PHB per cell for field-collected nodules was slightly wider in soya bean than in *C. fasciculata*, though differences in sample size curtail any direct comparison between these two groups. Our analysis of shoot biomass data from soya bean trap plants and *C. fasciculata* suggests that variation in plant size did not contribute significantly to variation in PHB per cell measured in nodules (electronic supplementary material, appendix C).

Assuming that structural (non-PHB) biomass is close to 0.2–0.5 pg cell^−1^ [57], the phenotypic distribution in figure 1 suggests that it is common for rhizobia in both host species to accumulate PHB as 50% or more of their biomass, which fits with previous studies on PHB accumulation in soya bean nodules [3,60]. For a cell with 0.5 pg of structural biomass, 2 pg of PHB would comprise 80% of its total biomass, which is within the range of PHB accumulation observed in multiple bacteria taxa grown in cultures [61]. The published estimate of PHB content we could find for symbiotic rhizobia was approximately 60%, found in *Bradyrhizobium* bacteroids inside soya bean nodules [60]. Our variation in our calibration standards suggests that our PHB measurements are less precise in the high end of our phenotypic distribution (i.e. near or above 1 pg cell^−1^; electronic supplementary material, appendix A). However, lesser precision in the high range does not negate the entire range of phenotypic variation captured by our measurements.

### 3.2. Heritable phenotypic variation in PHB accumulation

To what extent does the phenotypic variation in figure 1 represent genetic variation among rhizobia in the tendency to accumulate PHB, as opposed to non-heritable phenotypic variation (i.e. due to environmental variation) and measurement error? In split-root plants (figure 1*c*), the empirical 95% confidence interval for PHB per cell was nearly twice as large for nodules containing field-isolated rhizobia (variation due to genotype, environment and measurement error) as for nodules containing the reference strain (variation due to environment and measurement error only). Apparent broad-sense heritability (i.e. proportion of variance explained by isolate) was 0.63 (−0.06/+0.19) for the set of 20 field isolates measured in split-root plants (bootstrap mean ± 95% confidence interval with 10 000 resamples). Apparent broad-sense heritability was higher for the set of 20 isolates (plus the inoculum strain USDA110) tested in singly inoculated plants (0.83 + 0.08/−0.05, bootstrap mean ± 95% confidence interval with 10 000 resamples; electronic supplementary material, appendix D). As noted in Material and methods, heritability estimates may be augmented in singly inoculated plants due to stronger feedbacks between nitrogen fixation and total carbon supply. In both split-root and singly inoculated plants, the bottom of the 95% confidence interval was larger than 10 000 null heritability estimates produced by randomly reassigning plants to isolates, which rejects the null hypothesis that heritability estimates were a by-product of random variation among plant replicates.

Genetic variation in PHB accumulation was also apparent in our sample of *C. fasciculata* nodules, based on large differences in PHB accumulation associated with tolerance to 1 µg ml^−1^ BG (a taxonomically informative trait in *Bradyrhizobium* [45]). Estimated PHB per cell was lower and less-variable in the 14 nodules containing BG-sensitive rhizobia (mean = 0.043 ± 0.009 pg cell^−1^) compared to the 13 nodules containing BG-tolerant rhizobia (mean = 0.32 ± 0.035 pg cell^−1^) (bootstrap mean and standard error from 10 000 resamples). The difference in means between nodules with BG-sensitive and -tolerant rhizobia was statistically significant (*p* = 0.012) according to a two-sided permutation test with 10 000 permutations.

### 3.3. How does PHB accumulated during symbiosis affect the fitness of rhizobia?

Excessive diversion of resources from N fixation to PHB could trigger fitness-reducing host sanctions [62]. If so, then rhizobia could face trade-offs between fitness benefits of PHB in soil and fitness costs during symbiosis, depending on how much time they spend in each niche. Here, we focus only on the potential benefits of PHB for reproduction and survival in soil.

### 3.4. Model predictions

**Figure 2.**
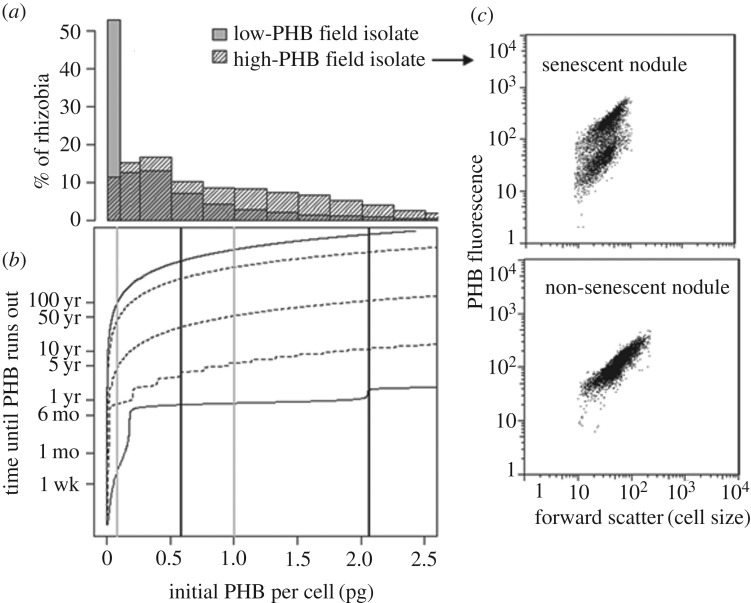
How long could PHB from nodules support survival in soil? (*a*) Measured distributions of PHB per cell for two field-isolated strains (approx. 3000 cells per strain pooled from six to eight senescent soya bean nodules from three host plants). (*b*) Model estimates for how long a rhizobia cell could support metabolism using only internal PHB, assuming starvation begins on 15 September. Oscillations within each curve represent changes in metabolic rate in response to seasonal changes in soil temperature. Estimated survival time decreases slightly if starvation begins in a warmer month (electronic supplementary material, appendix B). Solid curves represent full somatic maintenance and dormancy from Price & Sowers [22], while dotted curves are intermediate metabolic rates (10%, 1% and 0.1% of full somatic maintenance from bottom to top). Vertical lines show the geometric mean and 90th percentile PHB estimates for the low-PHB strain (grey) and the high-PHB strain (black) shown above in (*a*). (*c*) The top plot shows representative flow cytometry data from one of the senescent nodules used to obtain the distribution for the high-PHB isolate in (*a*), compared to data from the same isolate in a non-senescent nodule (bottom plot, not part of the dataset in (*a*)). Electronic supplementary material, appendix C contains further details on these data.

Our model predicts that PHB acquired in nodules could support rhizobial metabolism for several days to several centuries, depending on their metabolic state. While it has been suggested that rhizobia catabolize most or all of their PHB during nodule senescence [63], our measurements from senescent soya bean nodules suggest that rhizobia can retain substantial PHB when they exit symbiosis (figure 2*a*; electronic supplementary material, appendix C). Figure 2*b* shows how long PHB could support various metabolic rates within the range of reported values for non-growing prokaryotes (electronic supplementary material, appendix B [6]).

In a typical soya bean–corn rotation, rhizobia spend approximately 20 months in the soil between soya bean harvest and the next crop of soya bean plants. Our model estimates that a cell would require only 0.0009 pg PHB to support minimal dormancy for 20 months, which suggests that the average rhizobia cell from 94% of trap-plant soya bean nodules and 78% of field-collected soya bean nodules would have enough PHB to survive in a dormant state until the next soya bean crop and some for much longer (figure 1). Rhizobia with very high PHB could support even full somatic maintenance between soya bean crops, which would give them a potential competitive advantage against dormant cells [27].

Phenotypic variation within rhizobia strains complicates the question of how heritable differences in PHB accumulation contribute to fitness differences among rhizobia strains. Figure 2*a* shows the extent of PHB variation for two field isolates measured in soya bean nodules. Comparing their geometric means (the statistic we used to estimate PHB per cell in nodules, shown in figure 1), the average cell from the high-PHB isolate could apparently support nearly 10 months of full somatic maintenance, compared to only 13 days for the average cell of the low-PHB isolate. However, the upper 10% of cells from even the low-PHB isolate apparently had enough PHB to support 10 months of full somatic maintenance (approx. 1 pg PHB per cell). For a large soya bean nodule with 10^9^ rhizobia, the upper 10% of the phenotypic distribution in PHB per cell would represent 100 million cells that could potentially persist for much longer than the average for that nodule. These time estimates are not intended to exactly predict what happens in a natural setting, but to illustrate that non-heritable variability in PHB per cell among rhizobia cells from the same genotype could feasibly produce a wide variation in fitness during the free-living stage, potentially weakening natural selection due to heritable variation among genotypes.

Of course, it is important to acknowledge that measurement error probably contributes to the apparent phenotypic variation within nodules—including counts with unrealistically high PHB estimates that are probably bits of debris or clumped-together rhizobia missed by our gating procedure (electronic supplementary material, appendix A). Qualitative differences in flow cytometry data between senescent and non-senescent nodules containing the same rhizobia isolate (figure 2*c*) support our interpretation that the variation within isolates in figure 2*a* mostly represents true phenotypic variation and not just measurement error. Senescent nodules often showed two discrete clouds of PHB fluorescence—perhaps due to differences in the timing of senescence between inner and outer parts of the nodule [63]—whereas non-senescent nodules usually typically showed only one cloud of PHB fluorescence (figure 2*c* and electronic supplementary material, appendix C). It seems unlikely that random measurement error would produce such consistent, qualitative differences in the distribution of PHB fluorescence between senescent and non-senescent nodules.

### 3.5. Population trends in starving rhizobia

**Figure 3.**
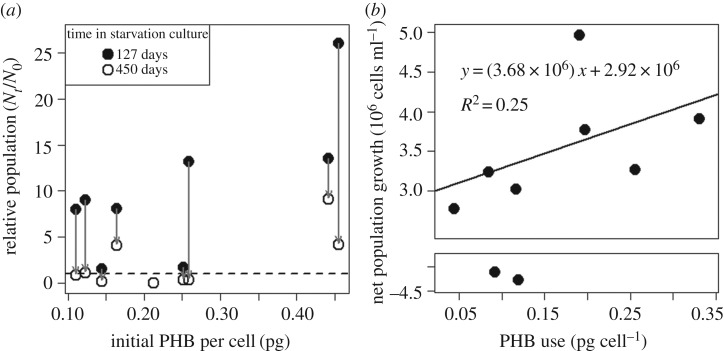
Initial PHB only partially explains population trends in starvation culture. Each point represents a different rhizobia isolate in starvation culture (mean of two to three replicate aliquots). (*a*) Relative population of viable cells (from plate counts) after 4 and 15 months of starvation. Grey arrows indicate changes for the same strain. Only three isolates retained higher-than-initial population size after 15 months of starvation. (*b*) Net population growth (starting from approximately 10^5^ CFU ml^−1^) versus PHB use during the first 29 days of starvation. The slope of the regression line (excluding two strains with net negative population growth) was not statistically significant from 0 (*p* = 0.25, *F* = 1.66 on 5 degrees of freedom).

Results from our nine rhizobia isolates with heritable variation in initial PHB per cell (acquired in soya bean nodules) are largely consistent with previous work with experimentally induced, within-strain PHB variation [8], showing that higher amounts of PHB enhanced the short-term growth and long-term persistence of *E. meliloti* populations in starvation culture [8]. Two of three isolates with the greatest population persistence (i.e. retaining higher-than-initial population size after 450 days of starvation) began with the highest PHB endowment (figure 3*a*). Overall, however, variable population growth and persistence among isolates with similar PHB endowment show that other factors contributed to population trends in starvation culture (figure 3*a*). A linear regression of PHB use versus short-term population growth (figure 3*b*) was not statistically significant (due to an outlier isolate) and the growth differences between isolates with the highest and lowest initial PHB was only approximately one-third of the extrapolated population growth without PHB (the y-intercept in figure 3*b*), apparently supported by external resources that were not eliminated during our washing and pyrolysing treatments. Interestingly, a previous study found similar levels of population growth for *Bradyrhizobium* in sterile water, even after extensive purification to remove organic contaminants [64].

### 3.6. PHB use in starving rhizobia

**Figure 4.**
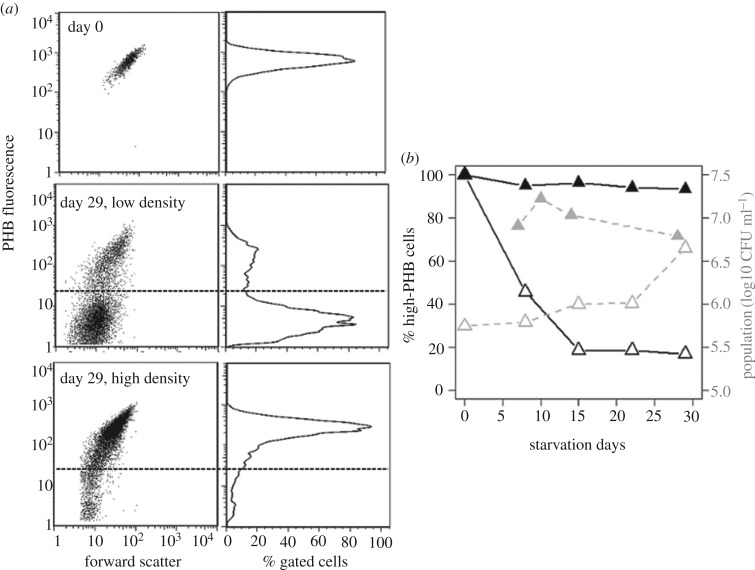
Segregation of high- and low-PHB subgroups in starvation cultures suggest asymmetric partitioning of PHB to old-pole cells. (*a*) Flow cytometry measurements (gated to exclude debris) for the same rhizobia population at different population densities (approx. 10^5^ or 10^6^ CFU ml^−1^), showing segregation at low density into high- and low-PHB subgroups (above and below dotted-line) after 29 days of starvation. (*b*) Trends in the proportion of cells in the high-PHB subgroup (solid lines) compared with viable population size (dashed lines) for the same population (each point is the mean of two to three replicate aliquots). Rhizobia starved at low cell density (open symbols) showed a decrease in the proportion of high-PHB cells concurrent with approximately 10-fold population growth, whereas the same population showed little-to-no PHB segregation or population growth at high density (filled symbols; day 0 data not available, but was diluted from the same culture as the low-density populations).

Bimodal PHB distributions in our starvation cultures, summarized in figure 4, support the hypothesis that our rhizobia isolates from soya bean nodules retain PHB mostly in the parental (old-pole) cell during cell division, as shown previously for *E. meliloti* 1021 [65]. Asymmetric partitioning of PHB into old-pole cells has been observed previously in *Bradyrhizobium* as well, based on microscopy with several strains [66].

Provisionally accepting that the high-PHB subgroup is made up of old-pole parental cells, as shown in *E. meliloti* [65] we can use changing levels of PHB per cell within this subgroup to infer how rhizobia use host-derived resources during starvation. This approach for measuring PHB use assumes that there is no growth in the high-PHB subgroup during starvation (i.e. due to PHB accumulation by daughter cells). Counts of gated cells in the high-PHB subgroup generally support this assumption (electronic supplementary material, appendix E), although there is no way to know for certain whether any PHB accumulation occurred in starvation cultures. We present results for one high-PHB isolate and one low-PHB isolate for clarity, although all nine *Bradyrhizobium* isolates showed a similar qualitative pattern of PHB use over time (electronic supplementary material, appendix E).

**Figure 5.**
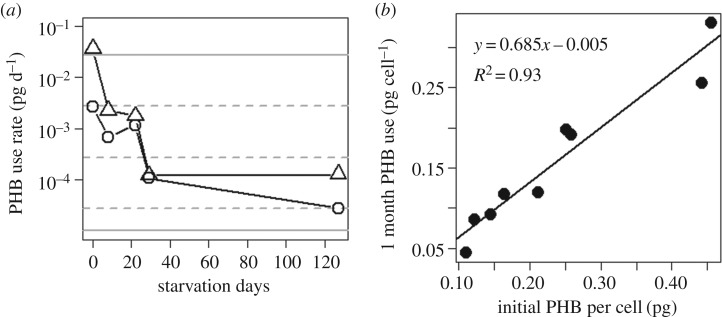
PHB use during starvation. (*a*) During the first month of starvation, the rate of PHB use slows in the high-PHB subgroup (i.e. ignoring low-PHB daughter cells, figure 4, and dividing the change in PHB in the high-PHB subgroup by the number of days elapsed, with final rates based on days 127–430). Two representative strains are shown: a high-PHB field isolate (triangles) and strain USDA110 (circles). Horizontal lines indicate theoretical PHB-use rates at 26°C (solid and dashed lines as in figure 2). (*b*) Rhizobia that acquired more PHB in nodules used more PHB during the first 29 days in starvation. Each point represents a different isolate in starvation culture (mean of two to three replicate aliquots).

PHB use per day (change in mean PHB per cell between sampling periods, divided by days elapsed) slowed rapidly during the first 30 days of starvation and stabilized thereafter (figure 5*a*). During the first week, the daily PHB-use rate was between 10% and 150% of the theoretical rate for full somatic maintenance (at 26°C with assumptions in electronic supplementary material, appendix B). Between month 1 and month 4, the daily PHB-use rate dropped to a small fraction of full somatic maintenance, remaining 10 times higher than minimal requirements for dormancy. These rhizobia apparently used approximately 70% of their initial PHB during the first few weeks of starvation before entering a dormant state.

Among isolates, the amount of PHB used during the first month in the starvation buffer correlated strongly with the initial amount acquired during symbiosis (figure 5*b*), so isolates that accumulated more PHB in soya bean nodules were apparently able to support a higher short-term metabolic rate. Once the metabolic rate slowed (as suggested by a smaller decrease in PHB per cell between sampling dates), all isolates maintained a portion of their initial PHB endowment for up to 127 days in starvation culture. After 15 months, only the two highest-accumulating isolates retained cells with a measurable fraction of their initial PHB endowment. The residual PHB in these two isolates suggests that PHB from nodules contributed to the survival of rhizobia for over a year in starvation culture. Our model predicts that the same amount of PHB would last even longer at temperatures typical of field soils, depending on the level of dormancy (figure 2).

## 4. Discussion

Through field and laboratory measurements, experiments and modelling, we laid the groundwork for understanding PHB accumulation as a life-history trait in symbiotic rhizobia. First, we showed that natural rhizobia populations contain considerable, apparently heritable, phenotypic variation in PHB accumulation in nodules. Second, we developed a more nuanced framework for understanding how variation in the amount of PHB stored during symbiosis contributes to variation in fitness after rhizobia are released from nodules. Rhizobia isolates from soya bean nodules (varying in PHB, but also, presumably, in other traits) did not show a clear relationship between the amount of PHB acquired in nodules and fitness in starvation culture, based on population growth and persistence over 15 months. However, patterns of PHB use in starving isolates suggest that PHB mediates fitness through a combination of short-term functions (directly or indirectly supporting reproduction) as well as long-term functions related to dormancy. Our model shows that rhizobia acquire enough PHB in nodules to support their metabolic needs for many years if they enter a dormant state. Therefore, the relationship between phenotypic variation in PHB accumulation and subsequent fitness in the soil will depend on how the ecological context shapes trade-offs between allocation to short- versus long-term functions.

### 4.1. Natural variation in PHB accumulation in nodules

We showed that co-occurring rhizobia interacting with an agricultural host (*G. max*) and a wild host (*C. fasciculata*) vary widely in the amount of PHB they acquire during symbiosis. Our experiments with *Bradyrhizobium* isolates in growth pouches suggest that this phenotypic variation in PHB accumulation was largely due to genetic differences among rhizobia. Although our heritability estimates from a single soya bean genotype do not incorporate variation due to host genotype, they represent the level of host genetic diversity in a typical soya bean planting. Measuring PHB with a lipid stain does not capture other potentially important storage molecules like glycogen [67]. However, previous results showed a correlation between PHB accumulation and overall carbon storage in a collection *Rhizobium* and *Ensifer* strains [68], so it is likely that variation in figure 1 reflects substantial variation in overall energy storage. Therefore, our results suggest that natural selection could act on phenotypic variation in PHB accumulation within rhizobia populations.

Previously, it has been proposed that positive directional selection for rhizobial PHB accumulation during the free-living stage could be counteracted by negative selection during symbiosis due to host sanctions against rhizobia that divert too much energy from N fixation [8,14]. Further research is needed to understand how the energetic trade-off between PHB accumulation and N fixation contributes to the conflict between legumes and rhizobia. The fact that all three samples of field-collected and trap-plant nodules contained rhizobia with very high PHB per cell (above approximately 0.5 pg) suggests that host sanctions do not prevent high-PHB rhizobia from persisting in natural populations. Considering that only a small fraction of the rhizobia population in soil enters into symbiosis even when hosts are abundant [69], fitness benefits of PHB during the free-living stage may contribute to the persistence of high-PHB accumulating rhizobia, even if sanctions constrain their reproduction inside nodules. One way to investigate how heritable variation in PHB accumulation among rhizobia contributes to fitness in and out of symbiosis would be to compare the phenotypic distribution of PHB among rhizobia populations that differ in the frequency of symbiotic interactions with legumes (e.g. due to varying intervals between host crops).

### 4.2. Implications for fitness

According to our model of PHB use under realistic soil temperatures and metabolic rates, a fully dormant rhizobia cell would require very little PHB to support its metabolic needs for the typical 20-month gap in availability of a soya bean host. While a high-PHB cell could potentially support dormant metabolism for centuries, we believe that a life-history strategy characterized by long-term dormancy supported by high resource acquisition would be unlikely to persist under natural selection. In particular, rhizobia that prolong survival by entering a state of deep dormancy could lag behind in responding to nodulation opportunities or to fluxes of resources released from plant roots [27], leading to eventual displacement by rhizobia that can respond to new resources more quickly (i.e. through higher resting metabolism). Rhizobia that store more PHB during periods of high resource availability (including symbiosis) might face a less severe trade-off between long-term survival and the ability to compete for short-term opportunities [70,71].

Our observations of rhizobia isolates in starvation culture suggest that PHB mediates rhizobial fitness through both short- and long-term functions. Isolates that accumulated more PHB per cell in soya bean nodules expended proportionally more PHB during their first month in starvation culture, similar to the pattern of PHB use previously shown in genetically identical *E. meliloti* populations varying in initial PHB per cell [8]. However, unlike *E. meliloti* populations, which converged to the same approximate PHB per cell during their first month in starvation buffer, each isolate in our study retained about 30% of its initial PHB endowment. So, even though the high-PHB isolates expended more on short-term functions (including reproduction), they still retained more PHB for long-term functions after transitioning to dormancy (based on PHB use rates in figure 5*b*). This pattern of PHB use suggests that differences in PHB accumulation among rhizobia strains could mediate fitness differences at multiple life-history stages, supporting reproduction when rhizobia are first released from nodules, plus long-term survival between hosts, and perhaps even competition to reach their next host.

Although variation in PHB accumulation among rhizobia isolates predicted short-term PHB use, the extent to which PHB enhanced reproduction in starvation cultures was unclear. Unlike genetically identical *E. meliloti* populations, which showed a strong, linear relationship between PHB use and short-term population growth in starvation culture [8], comparisons among our rhizobia isolates showed only a weak, non-significant relationship between PHB use and population growth (figure 3*b*). This result suggests that these isolates may vary in how they allocate stored PHB between reproduction and other short-term functions, such as motility. We also cannot be certain that short-term PHB use in starvation buffer represents short-term PHB use after leaving nodules. Perhaps moisture and trace nutrients in starvation buffer induce a ‘priming’ response, as observed in soils when water and trace nutrients induce microbes to emerge from dormancy and use internal resources to support metabolic activity [72].

Our starvation culture experiments suggest that population density influences how rhizobia allocate stored resources between reproduction and other functions. Population growth did not occur when the same isolates were starved at a 10-fold higher population density (figure 4), even though cells began with the same amount of PHB and showed a similar PHB-use pattern as in figure 5*a*. Concentrating resources in a single individual by not dividing (analogous to primogeniture) may improve the odds of persisting until a new host is available, whereas producing a greater number of low-quality offspring could lead to ‘boom or bust harvests of grandchildren’ [73]. A similar pattern of density-dependent population growth under starvation conditions was also found in a previous experiment with *Bradyrhizobium* populations in sterile water, which grew or shrunk up to 100-fold before converging to an apparent carrying capacity around 10^6^ CFU ml^−1^ [64], whose mechanistic basis (limiting resource) was unclear.

Even if host plants are present every year or two, nodulation opportunities for an individual rhizobia cell may occur at much longer intervals. Our model suggests that infrequent opportunities for symbiosis, along with strong competition for carbon sources in the rhizosphere, might favour high-PHB rhizobia strains that store large amounts of resources when they are available. The apparent evolutionary persistence of lower-PHB strains in natural rhizobia populations suggests that greater PHB accumulation sometimes has fitness costs, either during symbiosis or subsequently.

## Supplementary Material

Appendices A-E

## Data accessibility

Supporting analysis scripts and edited datasets have been archived in the Data Repository for the University of Minnesota (DRUM), where they can be accessed from https://doi.org/10.13020/D69H6N.
