# Supplementary material for: Resource acquisition and allocation traits in symbiotic rhizobia with implications for life-history outside of legume hosts
Source: R Soc Open Sci. 2018 Dec 12;5(12):181124. doi: 10.1098/rsos.181124 (PMC6304121; doi:10.1098/rsos.181124)
Supplement: Appendices A-E [file rsos181124supp1.pdf]

Appendix A: Additional detail on materials and methods 2
Appendix B: Further information on the metabolic model of PHB use 5
Appendix C: Additional results on genetic and environmental factors contributing to variation in rhizobial PHB accumulation 7
Appendix D: Within- and among-isolate PHB variation measured in singly-inoculated soybean plants. 10
Appendix E: Additional methods and results from starvation cultures. 11
References for Appendices A-E 14

Appendix A: Additional detail on materials and methods

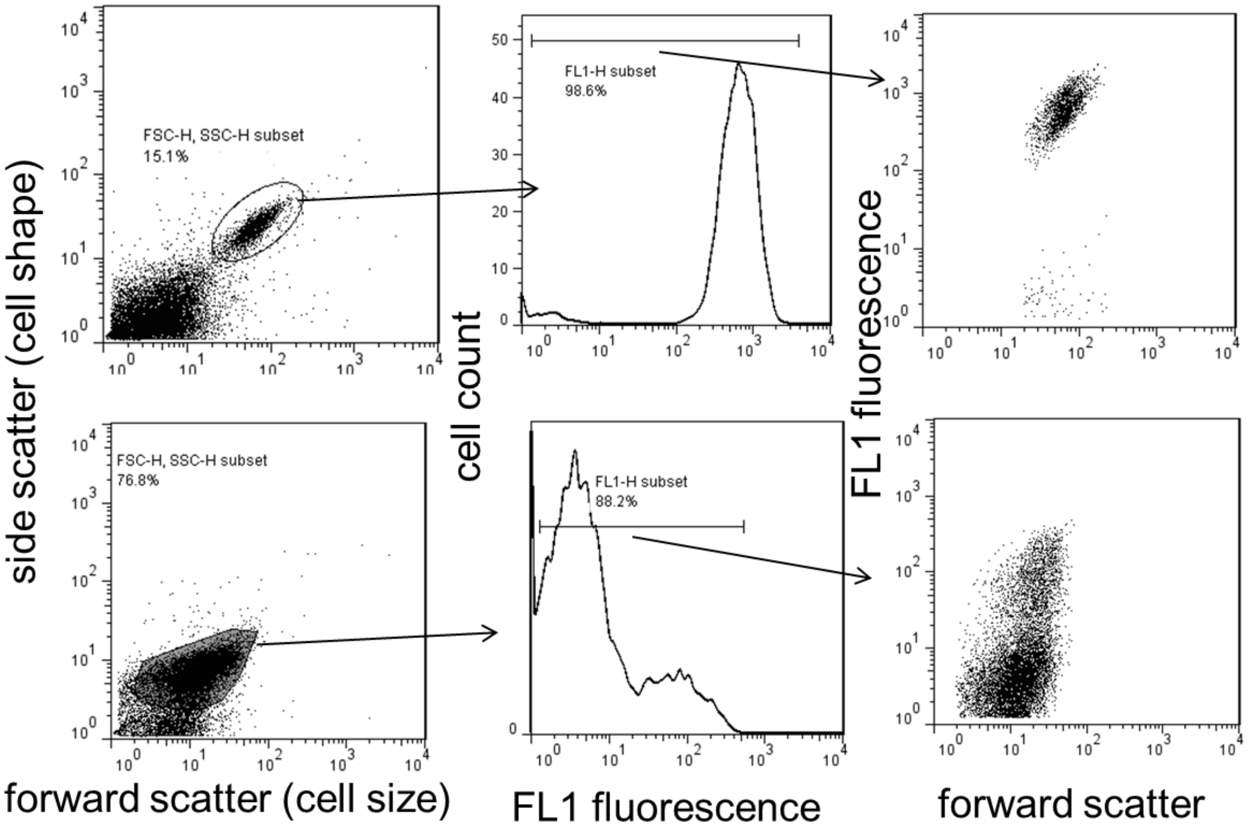

Figure A1. Gating to exclude debris from flow cytometry data.

Each row of panels represents the gating process for a single flow cytometry file. First, a region is selected based on forward and side scatter (left), then unstained particles are excluded based on a histogram of fluorescence, using FL1 fluorescence for the Bodipy stain or FL3 for the Nile Red stain (middle). The gated cell population (right) should show a positive correlation between PHB fluorescence and forward scatter (a proxy of cell size).

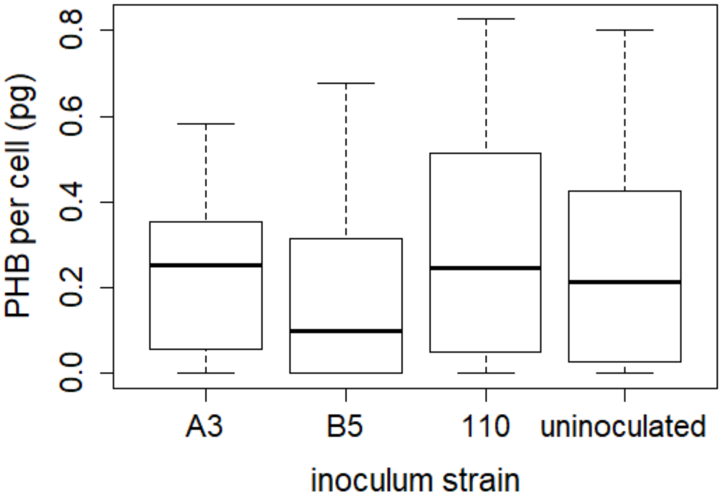

Figure A2: The distribution of PHB measurements in field-collected soybean nodules did not differ significantly between subplots from different inoculation treatments. The soybean nodules were sampled from 16 field-grown plants in Becker, MN that were originally part of an experiment intended to compare plant growth in response to four inoculation treatments: a high-PHB rhizobia isolate (A3, 21 nodules), a low-PHB field-isolate (B5, 23 nodules), a highly beneficial inoculum strain as a positive control (USDA110, 24 nodules), and sterile culture medium as an uninoculated negative control (uninoculated, 20 nodules).

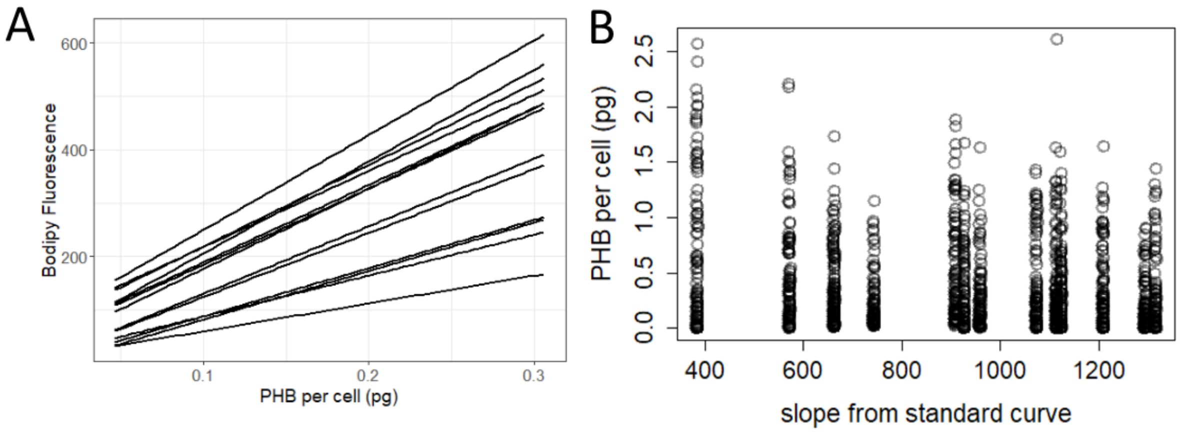

Figure A3: Effect of calibration error on PHB estimates. A) Variation in standard curves from *Ensifer meliloti* standards used to estimate mean PHB per cell in the sample of 1276 nodules shown in Fig. 1B of the main text. The same set of six standards was stained and measured with each batch of nodules measured on the flow cytometer (a total of 14 batches measured over ~1 month). Mean PHB per cell for nodules and standards was estimated as the geometric mean of Bodipy fluorescence (measured on the FL1 channel) for thousands of cells from a single sample. B) Measurement error due to variation in the slope of the standard curves from (A) may stretch the upper range of phenotypic variation measured in nodules (each point is the PHB estimate for a single nodule from the distribution shown in Fig. 1B of the main text).

Appendix B: Further information on the metabolic model of PHB use

Additional Background and rationale

Internal reserves of reduced carbon, such as PHB, can support metabolic functions that allow bacteria to survive and compete when external resources are scarce. The amount of carbon that a bacteria cell requires to support metabolic functions per unit time varies over a 10^6-fold range, depending on whether the cell is actively growing, maintaining somatic functions (e.g., protein turnover) without producing new biomass, or dormant (Morita 1997, Price and Sowers 2004). Temperature also affects a cell's carbon requirements, since the energy costs of somatic maintenance are higher at higher temperatures (Russell and Cook 1995). Price and Sowers (2004) used published measurements of prokaryote metabolism from various ecosystems to extrapolate functions for metabolic rate in response to temperature, with different parameters for prokaryotes that are growing, active but not growing, or dormant.

Rhizobia metabolize PHB by breaking down the PHB polymer ($[C_4H_6O_2]_n$) into a monomer of β-hydroxybutyrate ($C_4H_8O_3$), which is then oxidized into acetoacetate ($C_4H_6H_3$) and carried through the TCA cycle by the CoA enzyme (Lodwig and Poole 2003). Therefore, each atom of carbon in PHB represents an atom of reduced carbon that can support metabolism.

Price and Sowers (2004) defined two sets of metabolic parameters for nongrowing bacteria, based on distinct metabolic states proposed by Morita (1997). Full maintenance metabolism supports all cell functions aside from growth, such as protein turnover, motility, and osmotic regulation. Dormancy metabolism supports the minimal level of cell function needed to repair spontaneous damage to DNA and proteins that occurs in inactive cells. Bacteria recovered from permafrost show evidence of dormant metabolism supporting DNA repair for tens of thousands of years (Stewart Johnson et al. 2007). The energy for maintenance metabolism and DNA repair comes from reduced carbon entering the TCA cycle and exiting as CO_2. Therefore, metabolic rates were defined as the mass of reduced carbon needed to support the equivalent mass of structural biomass carbon per unit time (g C g^{-1} biomass C h^{-1}) (Price and Sowers 2004). Carbon stored in reserves like PHB does not add to the carbon cost of supporting structural biomass (Kooijman 2010).

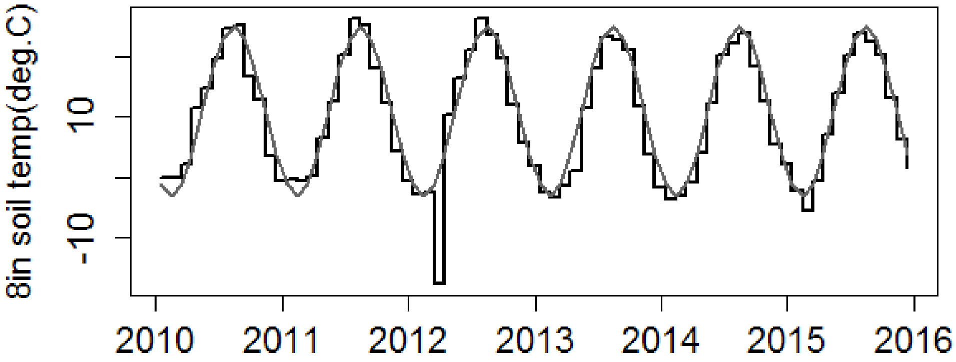

Figure B1: Seasonal oscillation in soil temperature. The black staggered line is the monthly average soil temperature, measured at a depth of 8 inches, from an agricultural research station in Waseca, Minnesota. The grey line is the mathematical approximation for seasonal temperature oscillation, presented in eq. 3.

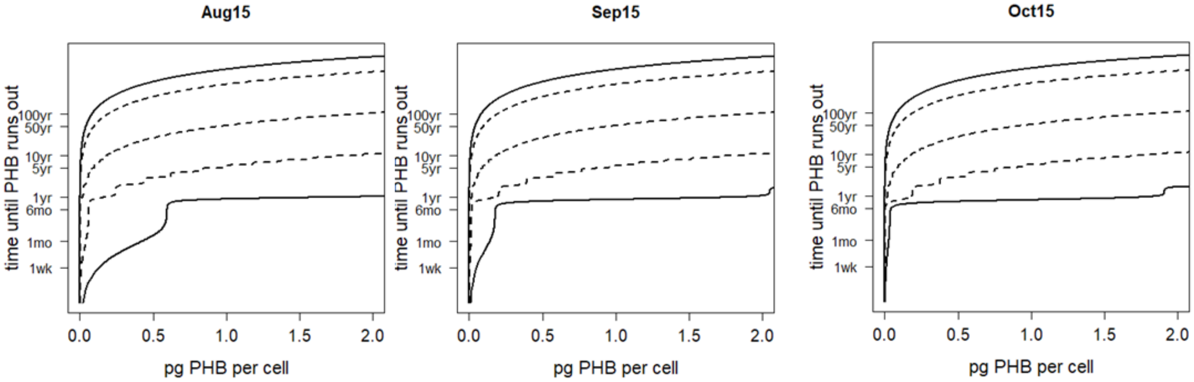

Figure B2: Effect of start date on survival estimates. Due to seasonal oscillation in soil temperature, shifting the start date by 1 month shortens (left) or prolongs (right) survival estimates, compared to those presented in Figure 2 (center), especially at higher metabolic rates.

Appendix C: Additional results on genetic and environmental factors contributing to variation in rhizobial PHB accumulation

Environmental sources of phenotypic variation

In addition to variation arising from genetic differences among rhizobia, variation in the amount of PHB that rhizobia accumulate during symbiosis can arise from environmental conditions within a nodule, among nodules within a host plant, and among host plants.

Environmental variation within a nodule:

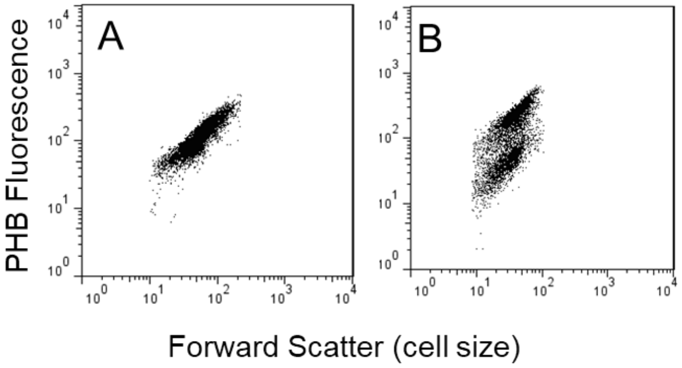

Figure C1: Nodule senescence increases rhizobial PHB variation within a nodule. Each flow cytometry diagram represents the same rhizobia strain paired with the same soybean genotype, extracted from a single nodule and stained with Nile Red, a quantitative lipid stain that serves as a proxy for PHB per cell. A) A representative nodule from a non-senescent plant (grown in a small growth pouch). B) A representative nodule from a senescent plant with dried pods and dehisced leaves (grown in 2 stacked Magenta units connected to give a greater volume of 1:1 sand:vermiculite substrate). Graphs exclude debris gated by forward and side scatter and FL3-fluorescence (Appendix A).

Environmental variation among nodules within a host plant:

Two factors that could create variation in rhizobial PHB among nodules within a host plant are differences in nodule age and unequal carbon allocation among nodules (e.g., in response to differences in nitrogen fixation). On singly-inoculated soybean plants, within-plant variation in nodule age (ranging from 16 to 30 days old) did not explain variation in rhizobial PHB among nodules (Fig. C2).

In field-collected and trap-plant nodules, unequal allocation among nodules likely contributed to phenotypic variation in PHB accumulation. Allocation differences could be random or they could reflect either absolute or relative sanctions. Under the relative sanctions

hypothesis (West et al. 2002), allocation to a nodule depends on a nodule's contribution relative to other nodules on the same plant. We would then expect the reference strain to accumulate more PHB on plants where it was paired with lower-quality focal strains. However, even though there was considerable phenotypic variation among nodules with the same reference strain (Fig. 1C in main text), PHB accumulation in the reference strain was little affected by which focal strain shared its host plant: variation among nodules paired with the same field isolate accounted for 97% of the total variance in PHB for the reference strain. These result seems inconsistent with the relative-sanctions hypothesis, although results could be different if differences among focal strains were greater.

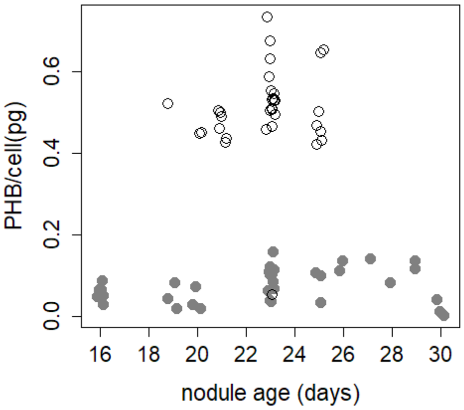

Figure C2: Rhizobial PHB accumulation did not show a strong relationship with nodule age, among nodules collected at the same time. Each point represents a nodule from a soybean plant inoculated with a single high-PHB field isolate (open circles) or low-PHB field isolate (filled grey circles) (3 plants per strain, 10-15 nodules per plant). Nodule age was tracked by marking appearance date on the outside of growth pouches. Nodules were harvested and fixed on the same date. These data suggest that differences in nodule age do not strongly contribute to variation in nodule PHB shown in Fig. 1 of the main text.

Environmental variation among host plants:

Environmental variation among plants could arise from variation in the total carbon supply to nodules due to differences in plant size, perhaps driven by differences among rhizobia strains in N fixation. In plants hosting a mixture of rhizobia strains (soybean trap plants and *C. fasciculata*), the amount of PHB per rhizobia cell, averaged across nodules, did not show a relationship with the shoot mass of the host plant (Fig. C3). This suggests that variation due to the plant's total carbon supply did not drive phenotypic variation in PHB accumulation shown in Fig. 1 of the main text. In fact, variation among host plants accounted for only 26% and 13% of variance in rhizobial PHB for field-collected and trap-plant soybean nodules, respectively. Nodules from the same *C. fasciculata* host plant also varied in PHB accumulation, although there were too few nodules per plant to estimate variance components (Appendix C). Overall, our measurements show that the majority of phenotypic variation in rhizobial PHB accumulation was driven by a combination of genetic variation among rhizobia and environmental variation among nodules on the same host plant.

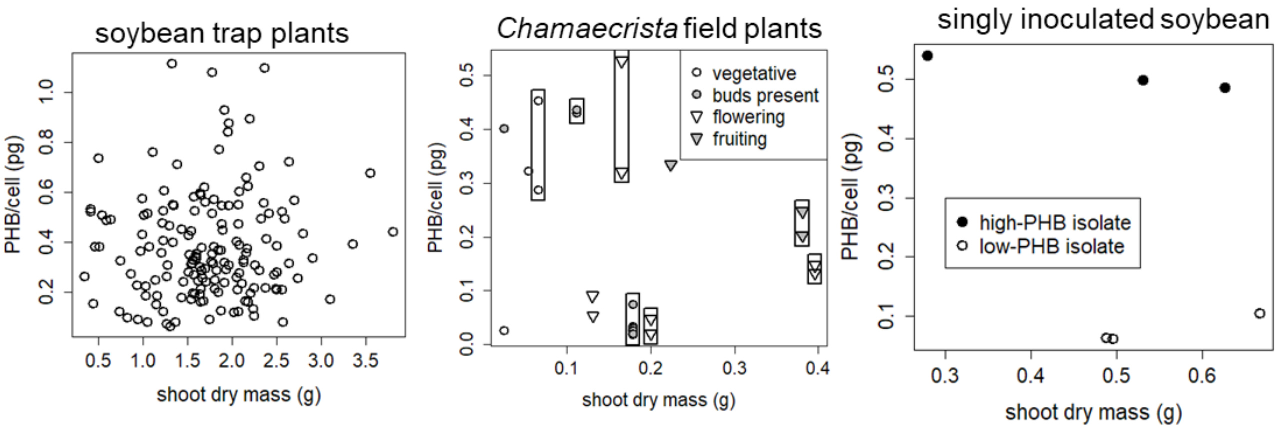

Figure C3: Rhizobial PHB accumulation did not show a strong relationship with plant size for hosts grown in a common environment.

For soybean trap plants inoculated with field-collected soil (left), each point represents shoot mass per plant and PHB/cell averaged across 8 nodules per plant. For *C. fasciculata* collected from the field (center), each point represents an individual nodule from host plants in the same field site. Boxes are drawn around nodules from the same host plant. For singly inoculated soybean plants in growth pouches (right), each point represents the mean PHB/cell for 9-15 nodules from a single plant.

Appendix D: Within- and among-isolate PHB variation measured in singly-inoculated soybean plants.

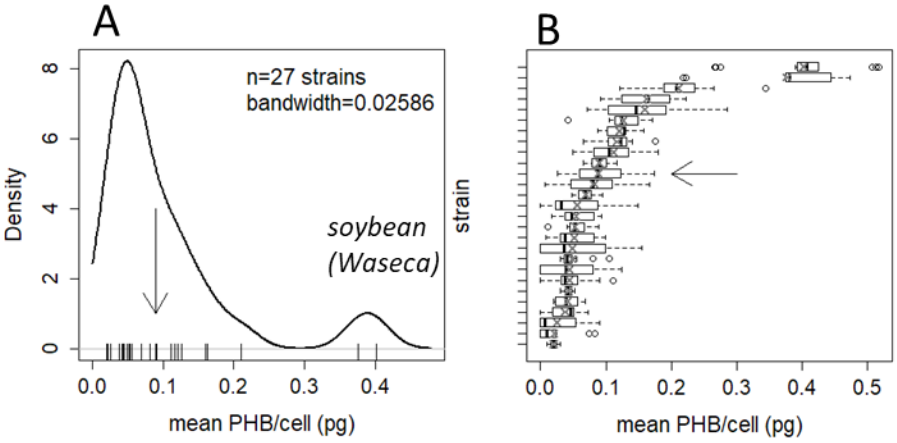

Figure D1. PHB variation in singly inoculated host plants. A) Variation in PHB accumulation among 27 rhizobia strains isolated from soybean nodules in Waseca, MN (except for the inoculum strain USDA110, which is marked with an arrow). Our starvation culture experiment used 9 of these strains, plus USDA110. Values for each strain are the mean PHB/cell across a total of 9 nodules sampled from 3 singly-inoculated soybean plants (MN0095, grown in pouches). B) Within-strain variation in PHB accumulation in the same soybean plants (same data as A, except replicate nodules are separate). Strains are sorted in order of increasing mean PHB/cell up the y-axis. The arrow indicates the inoculum strain USDA110.

Appendix E: Additional methods and results from starvation cultures.

Methods for setting up starvation cultures

Plate counts

During the first 3 weeks of sampling, we measured rhizobia population size with the “drop-plate” method (Chen et al. 2003), using 15-µL droplets to combine multiple dilutions on a single plate. Due to an increase in fast-growing contaminants in some samples, we switched to a spread-plate method at week 4, counting colonies in 100-µL dilutions spread over an entire Petri plate. Chen et al. (2003) found that both methods produce similar estimates.

Contaminants

Some aliquots had contaminants that could have been present inside surface-sterilized nodules or introduced during processing. One strain had to be excluded from the results because contaminants spread over dilution plates and covered rhizobia colonies. For the remaining nine strains, contaminants were distinguished from rhizobia based on colony morphology and faster appearance time than slow-growing *Bradyrhizobium*. Contamination in each aliquot was scored on a qualitative scale from 1 to 5 (1 = no contaminants, 2 = rhizobia greatly outnumber contaminants, 3 = approximately equal numbers of rhizobia and contaminants, 4 = contaminants moderately outnumber rhizobia, 5 = contaminants greatly outnumber rhizobia). Contamination did not appear to have a major impact on rhizobial survival, based on a comparison of rhizobia counts among replicate aliquots differing in contamination. Removing replicates with substantial contamination (contamination level 3-5) removes most of our data and does not qualitatively change results (Fig. E1, E2). Therefore, we presented results from all replicates with countable rhizobia, regardless of the level of contamination, acknowledging that contaminants likely add noise.

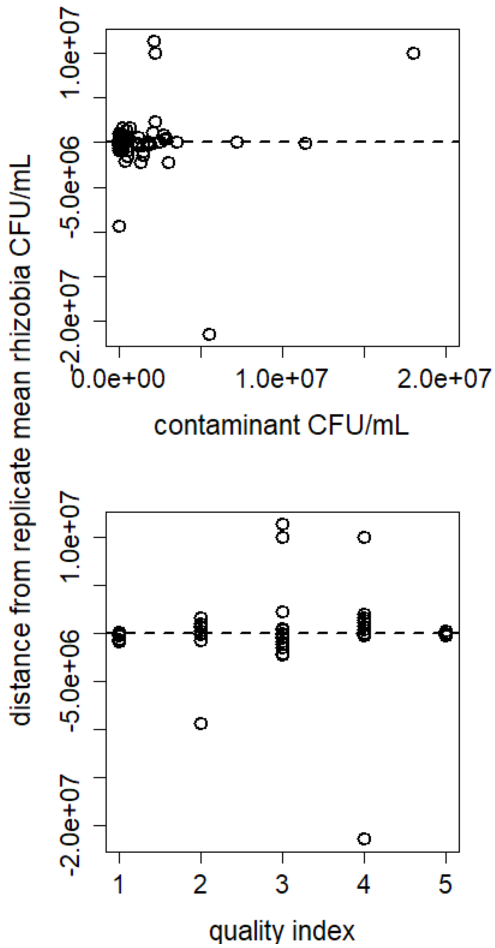

Figure E1: Contaminants in starvation cultures. Contamination did not appear to affect starvation survival among replicate aliquots varying in contaminant CFU/mL. The y-axis shows distance from the replicate mean CFU/mL for each replicate aliquot of the same strain sampled at the same time. The horizontal line marks zero (no change from replicate mean). Contaminant colonies were distinguishable

from rhizobia colonies based on appearance and faster growth. Contamination was scored in two ways: as a count of colonies (upper plot) and as a quality index (lower plot): 1= no contamination, 2 = slight contamination (a few contaminant colonies), 3 = moderate contamination (contaminant colony count < = rhizobia colony count), 4 = heavy contamination (contaminant colony count > rhizobia colony count), 5 = severe contamination (too many colonies to count, or large colonies spread over entire Petri plate).

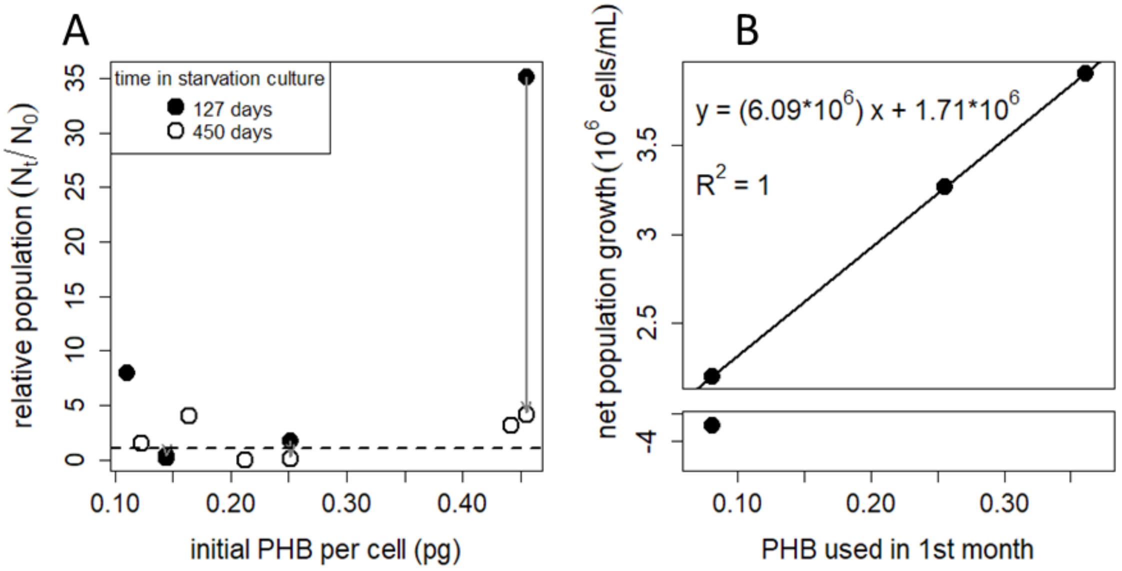

Figure E2: Removing contaminated samples does not qualitatively change population trends in starvation culture. A and B correspond to Fig. 3A and 3B from the main text, excluding aliquots of starvation culture that had a contamination index greater than 2 (see Fig. E1). Net population growth is the population size after 29 days of starvation minus initial population size (both estimated with dilution plating). Gray arrows in A connect observations from the same strain at 127 and 450 days in starvation culture.

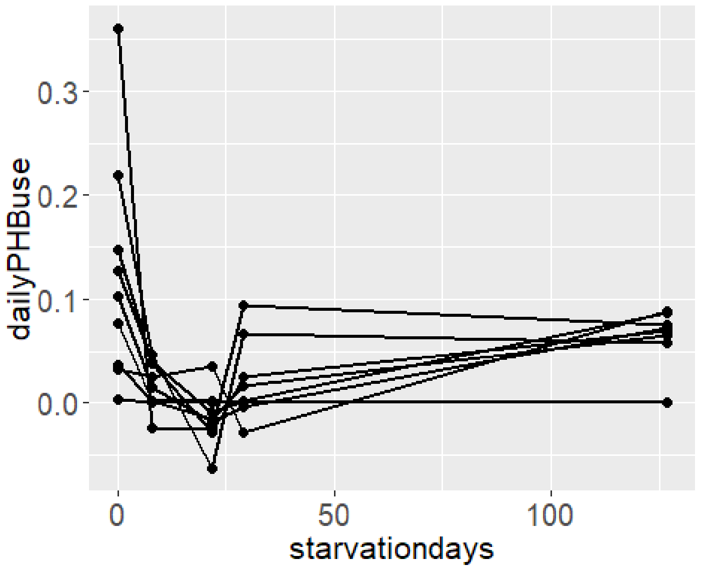

Figure E3: Change of PHB use over time for 9 strains (2 used in Fig. 5A of the main text)

PHB-use rate (pg/day) was estimated by dividing decrease in PHB between sampling periods by the number of days elapsed. PHB measurements exclude putative new-pole offspring produced in starvation buffer (i.e. the

low-PHB subgroup illustrated in Fig. 4). The final PHB-use rate was calculated from aliquots fixed at 127 and 450 days of starvation.

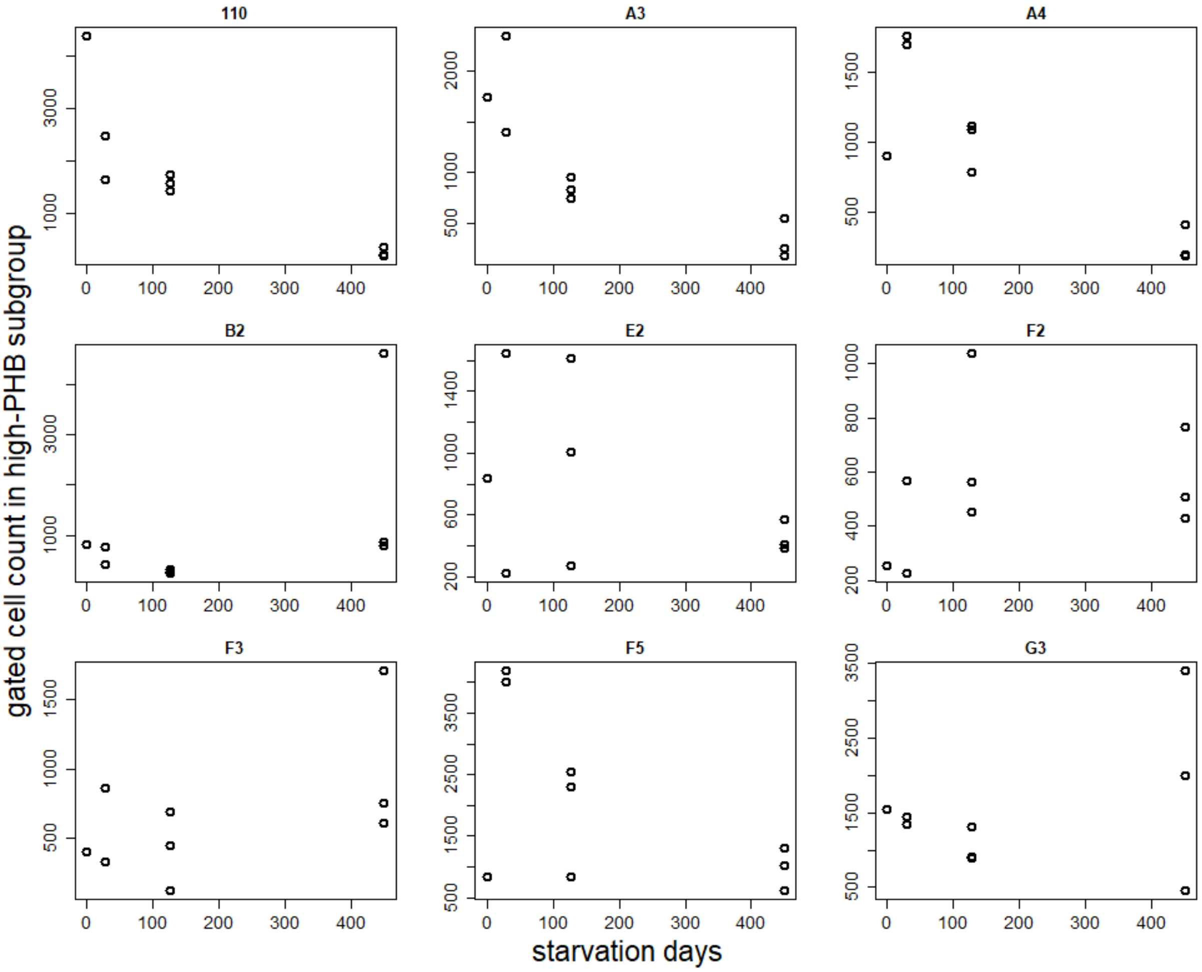

Fig. E4: Gated cell counts in the high-PHB subgroup from flow cytometry. Each panel depicts a different rhizobia isolate or strain (110 = *Bradyrhizobium diazoefficiens* USDA110). Figure 4 of the main text describes the process for gating high- and low-PHB subgroups. The cell count is imprecise due to problems with the flow rate mechanism in the flow cytometer and overlap between high- and low-PHB subgroups in rhizobia strains with lower initial PHB per cell.

References for Appendices A-E

Kooijman, S. A. L. M. 2010. Dynamic Energy Budget Theory for Metabolic Organization. Third edition. Cambridge University Press.

Lodwig, E., and P. Poole. 2003. Metabolism of *Rhizobium* bacteroids. Critical Reviews in Plant Sciences 22:37–78.

Morita, R. Y. 1997. Bacteria in Oligotrophic Environments: Starvation Survival Lifestyle. Chapman & Hall.

Price, P. B., and T. Sowers. 2004. Temperature dependence of metabolic rates for microbial growth, maintenance, and survival. Proceedings of the National Academy of Sciences 101:4631–4636.

Stewart Johnson, S., M. B. Hebsgaard, T. R. Christensen, M. Mastepanov, R. Nielson, K. Munch, T. Brand, M. T. P. Gilbert, M. T. Zuber, M. Bunce, R. Ronn, D. Gilichinisky, D. Froese, and E. Willerslev. 2007. Ancient bacteria show evidence of DNA repair. Proceedings of the National Academy of Sciences 104:14401–14405.

West, S. A., E. T. Kiers, E. L. Simms, and R. F. Denison. 2002. Sanctions and mutualism stability: Why do rhizobia fix nitrogen? Proceedings of the Royal Society B 269:685–694.
